# IL‐17A promotes the invasion–metastasis cascade via the AKT pathway in hepatocellular carcinoma

**DOI:** 10.1002/1878-0261.12306

**Published:** 2018-04-26

**Authors:** Qing‐guo Xu, Jian Yu, Xing‐gang Guo, Guo‐jun Hou, Sheng‐xian Yuan, Yuan Yang, Yun Yang, Hui Liu, Ze‐ya Pan, Fu Yang, Fang‐ming Gu, Wei‐ping Zhou

**Affiliations:** ^1^ The Third Department of Hepatic Surgery Eastern Hepatobiliary Surgery Hospital Second Military Medical University Shanghai China; ^2^ Key Laboratory of Signaling Regulation and Targeting Therapy of Liver Cancer (SMMU) Ministry of Education Shanghai China; ^3^ Shanghai Key Laboratory of Hepatobiliary Tumor Biology (EHBH) China; ^4^ The Department of Medical Genetics Second Military Medical University Shanghai China

**Keywords:** colonization, epithelial–mesenchymal transition, hepatocellular carcinoma, interleukin‐17A, secukinumab

## Abstract

We previously demonstrated that interleukin‐17A (IL‐17A) is associated with the progression of hepatocellular carcinoma (HCC). However, its role in the invasion–metastasis cascade of HCC and the efficacy of IL‐17A‐targeting therapeutics in HCC remain largely unknown. In this study, we found that IL‐17A promoted intrahepatic and pulmonary metastasesis of HCC cells in an orthotopic implant model. Moreover, our results showed that IL‐17A induced epithelial–mesenchymal transition (EMT) and promoted HCC cell colonization *in vitro* and *in vivo*, and the role of IL‐17A in invasion–metastasis was dependent on activation of the AKT pathway. Remarkably, combined therapy using both secukinumab and sorafenib has better inhibition on tumour growth and metastasis compared to sorafenib monotherapy. Additionally, the combination of intratumoral IL‐17A+ cells and E‐cadherin predicted the outcome of patients with HCC at an early stage after hepatectomy based on tissue microarray and immunohistochemistry. In conclusion, our studies reveal that IL‐17A induces early EMT and promotes late colonization of HCC metastasis by activating AKT signalling. Secukinumab is a promising candidate for clinical development in combination with sorafenib for the management of HCC.

**Abbreviations**
BCLC staging: Barcelona clinic liver cancer staging
EMT: epithelial–mesenchymal transition
HCC: hepatocellular carcinoma
IL‐17A: interleukin‐17A
IL‐6: interleukin‐6
MAH: metastasis‐averse HCC
MET: mesenchymal–epithelial transition
MIH: metastasis‐inclined HCC
OS: overall survival
PVTT: portal vein tumour thrombus
RFS: recurrence‐free survival
TGF‐β: transforming growth factor‐β
Th17: T‐helper cell
TMA: tissue microarray

## 1 Introduction

Hepatocellular carcinoma (HCC) is one of the most common and aggressive human malignancies in the world (Torre *et al*., 2015). Although successful partial hepatectomy has significantly improved survival, the prognosis of HCC remains poor because of tumour invasiveness, frequent intrahepatic spread and extrahepatic metastasis (European Association for The Study of The Liver; European Organisation For Research and Treatment Of Cancer, 2012). Elucidation of the molecular mechanisms underlying the HCC metastatic cascade is of utmost importance for the development of future strategies for treating HCC.

The tumour invasion–metastasis cascade is a stepwise and multistage process in which tumour cells disseminate from primary sites and spread to form colonies at distant sites through the systemic haematogenous or lymphatic circulation (Jin *et al*., 2017; Valastyan and Weinberg, 2011). In epithelial cancer, the epithelial–mesenchymal transition (EMT) plays a crucial role in the early events of tumour cell metastatic dissemination, which occurs when cells lose cell–cell contacts and acquire increased motility to spread into surrounding or distant tissues (Nieto *et al*., 2016; Thiery *et al*., 2009). The downregulation of E‐cadherin, a cell‐to‐cell adhesion molecule, and upregulation of mesenchymal markers (e.g. N‐cadherin and vimentin) are considered hallmarks of EMT (Zeisberg and Neilson, 2009). As a late step of metastasis, colonization determines whether the disseminated carcinoma cells will succeed in forming significant metastases (Pachmayr *et al*., 2017). The reverse process of EMT, the mesenchymal–epithelial transition (MET), is associated with the tumour‐initiating ability required for metastatic colonization (Ocana *et al*., 2012). Although there is much accumulated evidence showing the relevance of EMT and metastatic colonization for HCC prognosis during the past decade (Dou *et al*., 2017; Giannelli *et al*., 2016), the complicated mechanisms of HCC metastasis remain elusive.

Hepatocellular carcinomais usually secondary to inflammatory conditions due to chronic hepatitis and cirrhosis resulting either from hepatitis B/C virus infection or from non‐viral‐related causes, such as alcohol or obesity (Forner *et al*., 2012). Several inflammatory molecules, such as transforming growth factor‐β (TGF‐β) and interleukin‐6 (IL‐6), favour the invasion–metastasis cascade of HCC (Reichl *et al*., 2012; Wang *et al*., 2016). Interleukin‐17A (IL‐17A), the production of which characterizes a subset of CD4+ helper T cells (Th17 cells), has been implicated in certain tumours, affecting tumorigenesis, proliferation and angiogenesis (Song and Yang, 2017). Previously, we demonstrated that IL‐17A was associated with HCC prognosis and promoted tumour progression (Gu *et al*., 2011). However, the specific roles of IL‐17A in regulating EMT and its impact on the metastatic colonization of HCC cells are not well studied. In this study, we investigated the direct role of IL‐17A on the invasion–metastasis cascade of HCC to elucidate the underlying mechanism by specifically focusing on EMT and colonization.

## 2 Materials and methods

### 2.1 Clinical samples and follow‐up

All hepatic specimens were obtained from individuals who underwent surgical resection in the Eastern Hepatobiliary Surgery Hospital (EHBH; Shanghai, China). The study was approved by the Institutional Review Board of Eastern Hepatobiliary Surgery Hospital (Shanghai, China). All patients gave their written informed consent to participate in the study.

This study included 80 HCC specimens [including 40 metastasis‐inclined HCC (MIH) and 40 metastasis‐averse HCC (MAH)]. The MIH and MAH status was defined as described previously (Budhu *et al*., 2006; Ye *et al*., 2003). MIH refers to tumour tissues from patients with solitary HCC accompanied by portal vein metastasis or venous metastases or who developed distant metastases that were confirmed at follow‐up; MAH refers to tumour tissues from patients with solitary HCC and no detectable metastases at the time of diagnosis and at follow‐up. Paired tumour and portal vein tumour thrombus (PVTT) tissues from 30 patients with HCC were also included. The tissue microarray (TMA) containing 313 HCC patient samples (randomly collected from January 2006 to September 2010) was used to test the expression levels and clinical significance of IL‐17A. The clinical characteristics of the TMA were described previously (Tao *et al*., 2015). The follow‐up was performed as described previously (Tao *et al*., 2015). All tissues were preserved at −80 °C until use.

### 2.2 Cell lines

The HCC cell lines HCCLM6 and Huh7 were obtained from the Chinese Academy of Sciences Cell Bank. The patient‐derived primary HCC cell line HCC1664 was acquired as described previously (Ma *et al*., 2017). Cells were cultured in Dulbecco's modified Eagle's medium supplemented with 10% FBS and maintained in a 37 °C incubator with an atmosphere of 5% CO_2_.

### 2.3 Animal studies

The animal studies were approved by the Institutional Animal Care and Use Committee of Second Military Medical University (Shanghai, China). Male athymic BALB/c nude mice (5 weeks old) were used for animal studies.

### 2.4 The orthotopic implant model

A total of 1 × 10^7^ HCCLM6 or Huh7 cells labelled with GFP were subcutaneously implanted into the bilateral armpit of two BALB/c nude mice, respectively. Mice were sacrificed 4 weeks later, and pieces of tumour tissue were orthotopically grafted into the livers of nude mice to establish a GFP‐labelled, orthotopic HCC model. To evaluate the effects of IL‐17A in the models, mice (*n* = 6/group) were treated with 7 weekly doses of IL‐17A (1 μg·mouse^−1^ per day, intraperitoneally) or PBS (control) starting 1 day after implantation. Then, the mice were euthanized. Intrahepatic metastasis foci were counted by naked eye, and pulmonary metastasis foci were measured by haematoxylin/eosin staining.

To assess the efficacy of the secukinumab/sorafenib combination in preventing HCC metastases, the orthotopically implanted tumours were allowed to grow for 2 weeks. Mice were then randomized into three groups (*n* = 6/group): IL‐17A (1 μg·mouse^−1^ per day, intraperitoneally), IL‐17A + sorafenib (30 mg·kg^−1^ per day, orally), or IL‐17A + sorafenib + secukinumab (500 μg·mouse^−1^ per week, intraperitoneally), for 5 consecutive weeks. Then, we euthanized the mice and measured the size and frequency of the intrahepatic tumours.

### 2.5 *In vivo* colonization assay

We investigated the effect and mechanism of IL‐17A on intrahepatic and pulmonary tumour colonization. BALB/c nude mice were injected with 5 × 10^6^ HCCLM6 or Huh7 cells in 150 μL serum‐free MEM into the portal vein through the splenic hilum or into the tail vein. HCCLM6 cells were previously transfected with pCMV‐luciferase and selected with neomycin (800 μg·mL^−1^). To explore the influence of IL‐17A on metastatic colonization in the liver, mice (*n* = 5/group) were treated with IL‐17A (1 μg·mouse^−1^ per day, intraperitoneally) or PBS (control). To investigate whether AKT phosphorylation mediated the effects of IL‐17A on colonization, mice were then randomized into two groups (*n* = 5/group): IL‐17A (1 μg°mouse^−1^ per day, intraperitoneally) or IL‐17A + MK2206 (60 mg·kg^−1^ per 2 days, orally). To test the contribution of IL‐6 to the pro‐colonization role of IL‐17A, mice were then randomized into three groups (*n* = 5/group): IL‐17A (1 μg·mouse^−1^ per day, intraperitoneally) or IL‐17A + anti‐IL‐6 mAb (1 mg·mouse^−1^ per week, intraperitoneally) or IL‐17A + MK2206 (60 mg·kg^−1^ per 2 days, orally). The metastases of HCCLM6 cells were detected using the IVIS@ Lumina II system (Caliper Life Sciences, Hopkinton, MA, USA) 10 min after intraperitoneal injection of 4.0 mg luciferin (Gold Biotechnology, St. Louis, MO, USA) in 50 μL of saline. Mice were sacrificed 6 weeks later.

### 2.6 Statistical analysis

All statistical analyses in this study were performed with spss 18.0 software (IBM SPSS, Chicago, IL, USA). The chi‐square test or Fisher's exact test was used to compare qualitative variables, and Student's *t*‐test or the Mann–Whitney test was used to compare continuous variables. Pearson correlation analysis was performed to determine the correlation between two variables. Kaplan–Meier analysis and log‐rank test were used to evaluate the differences in patient survival. Cox proportional hazards regression analysis was used to analyse the effect of clinical variables on patient survival based on variables selected after the univariate analysis. A *P* value < 0.05 was considered significant. Detailed description of Material and methods can be found in the Appendix S1. The primers used in study were listed in Table S2.

## 3 Results

### 3.1 IL‐17A promotes HCC metastasis and is associated with EMT markers

**Figure 1:**
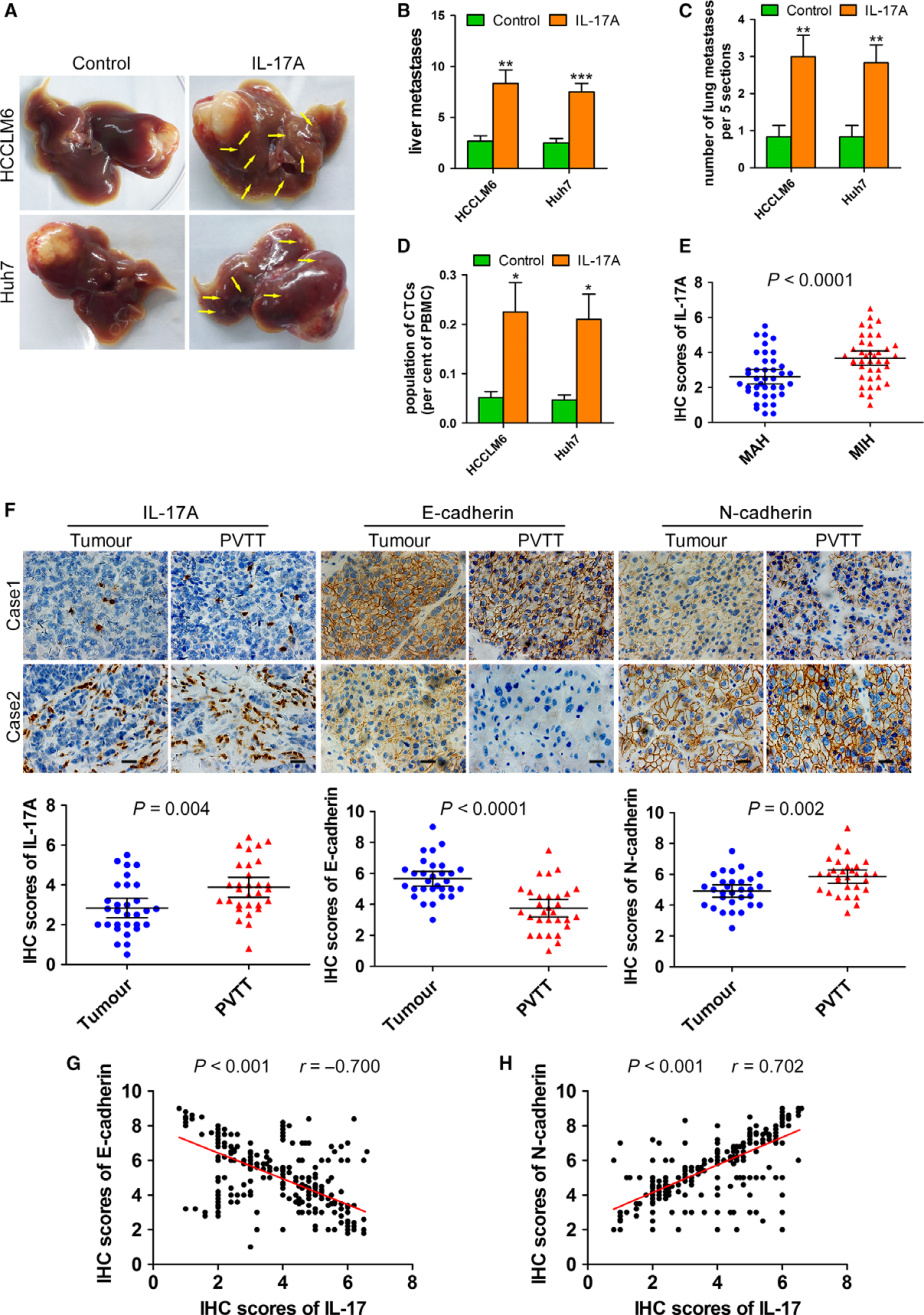
IL‐17A is involved in HCC metastasis and related to EMT markers. (A–D) An orthotopic implantation tumour model was established using HCCLM6 or Huh7 cells. Mice (*n* = 6/group) were treated with seven weekly doses of IL‐17A (1 μg per day, intraperitoneally) or PBS (control) starting 1 day after implantation. (A) Representative livers from orthotopically implanted mice. Yellow arrows: metastatic foci. (B) Number of liver metastases in mice from (A). (C) Number of metastatic nodules in the lungs from orthotopically implanted mice (five sections evaluated per lung). (D) The number of GFP‐labelled CTCs was examined by flow cytometry from whole‐blood samples of orthotopically implanted mice. (E) Immunohistochemistry of IL‐17A in 40 MIH and 40 MAH tumour tissues. (F) Immunohistochemistry of IL‐17A, E‐cadherin and N‐cadherin in 30 primary tumour tissues and matched PVTT tissues. Scale bars = 20 μm. (G‐H) Immunohistochemistry of IL‐17A, E‐cadherin and N‐cadherin on the TMA that included 313 primary tumour tissues. The correlation between IL‐17A and E‐cadherin (G) and IL‐17A and N‐cadherin (H) was measured by Pearson correlation analysis. All data are shown as mean ± SD. *: *P* < 0.05, **: *P* < 0.01, ***: *P* < 0.001, compared with the control group.

To evaluate the effects of IL‐17A on *in vivo* metastasis, we established an orthotopic implantation tumour model to test HCC cell invasion and metastasis. HCCLM6 and Huh7 cells were inoculated subcutaneously into nude mice, and the subcutaneous tumour tissues were used to establish orthotopic tumour models after 4 weeks. In this model system, the incidence and number of both intrahepatic and pulmonary metastases in the IL‐17A‐injected group was significantly increased compared with the control group (Fig. 1A–C). As both the HCCLM6 and Huh7 cells were labelled with GFP, we examined the number of circulating tumour cells (CTCs) from whole‐blood samples by flow cytometry and found that IL‐17A stimulation significantly increased the number of CTCs (Fig. 1D). Then, we performed immunohistochemical analysis of 80 tumour tissues from patients with HCC who underwent surgical resection at EHBH, including 40 MIH and 40 MAH. The expression level of IL‐17A was quantified on the basis of a multiplicative index of staining extent (0–3) and the average staining intensity (0–3). The percentage of IL‐17A‐producing (IL‐17A+) cells was significantly higher in the MIH than in the MAH (Fig. 1E). PVTT is the main route for metastasis in patients with HCC. We detected the percentage of IL‐17A+ cells in 30 cases of PVTT and matched primary tumour tissues collected from patients with HCC who underwent surgical resection at EHBH. A higher percentage of IL‐17A+ cells was observed in PVTT than in tumour tissues (Fig. 1F).

Epithelial–mesenchymal transition plays a vital role in the early events of tumour cell invasion and metastasis. Therefore, we investigated whether the EMT markers of HCC cells were associated with IL‐17A in clinical samples. Immunohistochemical analysis of the epithelial marker E‐cadherin and the mesenchymal marker N‐cadherin in 30 PVTT and matched primary tumour tissues revealed that in PVTT tissues, the expression level of E‐cadherin was reduced, and the expression level of N‐cadherin was upregulated compared with that in tumour tissues (Fig. 1F). Remarkably, the percentage of IL‐17A+ cells was inversely correlated with the E‐cadherin level and positively related to the N‐cadherin level in both PVTT and primary tumour tissues (Fig. S1A–D). Additionally, immunohistochemical analysis of IL‐17A, E‐cadherin and N‐cadherin on the TMA that included 313 HCC tumour specimens from patients with HCC who underwent surgical resection at EHBH revealed consistent results (Figs 1G,H and S1E) (Tao *et al*., 2015). Taken together, these results demonstrate that high levels of IL‐17A are associated with metastatic HCC and EMT markers.

### 3.2 IL‐17A induces EMT in HCC cells

**Figure 2:**
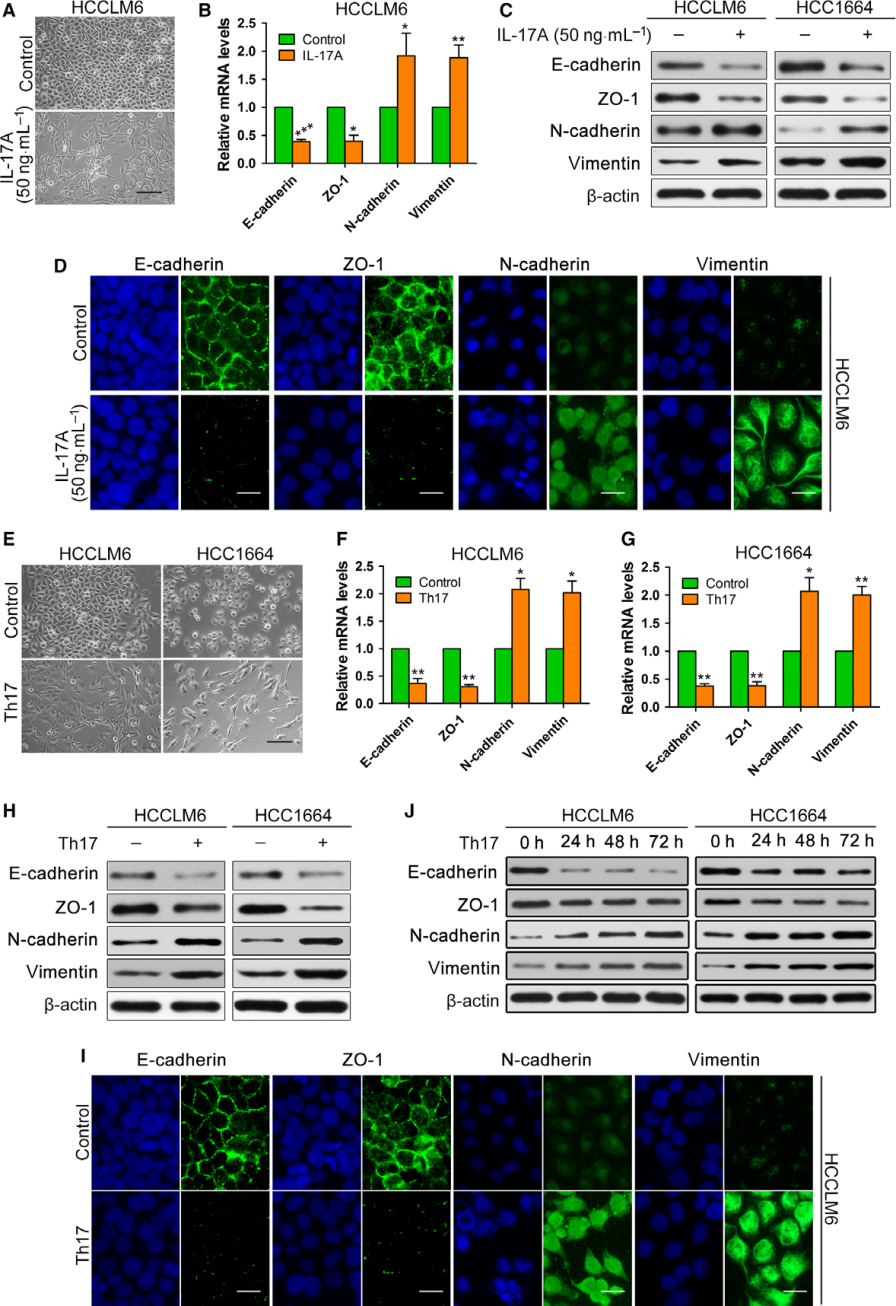
Effects of IL‐17A on EMT of HCC cells *in vitro*. (A–D) HCCLM6 cells or HCC1664 cells were stimulated continuously with exogenous IL‐17A (50 ng·mL^−1^) or PBS (control) for 2 weeks. (A) Phase‐contrast micrographs of the indicated HCCLM6 cells. Scale bars = 100 μm. (B,C) The mRNA (B) or protein (C) levels of EMT markers in the indicated HCCLM6 cells or HCC1664 cells. (D) Immunofluorescence microscopy analysis of the localization and expression of EMT markers in HCCLM6 cells. Scale bars = 50 μm. (E–I) Conditioned culture media from Th17 cells were collected and individually added into the medium of HCCLM6 or HCC1664 cells for 2 weeks. (E) Phase‐contrast micrographs of the indicated HCCLM6 or HCC1664 cells. Scale bars = 100 μm. (F–H) The mRNA (F,G) or protein (H) levels of EMT markers in the indicated HCCLM6 cells or HCC1664 cells. (I) Immunofluorescence microscopy analysis of the localization and expression of EMT markers in HCCLM6 cells. Scale bars = 50 μm. (J) Western blotting showed that EMT markers were changed as early as 24 h after treatment with culture medium from Th17 cells. All data are shown as mean ± SD. *: *P* < 0.05, **: *P* < 0.01, ***: *P* < 0.001, compared with the control group.

To further define the role of IL‐17A in the EMT of HCC cells, we first examined the effect of IL‐17A on cell phenotypes. We treated HCCLM6 cells and HCC1664 cells (patient‐derived primary HCC cell) expressing the IL‐17A receptor with exogenous IL‐17A (50 ng·mL^−1^) for 2 weeks (Fig. S2A), which caused the cells to undergo EMT, as indicated by a spindle ‐shaped appearance (Figs 2A and S2B). qRT‐PCR, western blotting, and immunofluorescence assays indicated that exogenous IL‐17A significantly decreased the expression of epithelial markers (E‐cadherin and ZO‐1) and increased the expression of mesenchymal markers (N‐cadherin and vimentin) in HCCLM6 cells, HCC1664 cells and Huh7 cells (Figs 2B–D and S2C–F). We also found that IL‐17A increased the expression of EMT‐transcription factors, including snail, slug and twist1, in HCCLM6 and Huh7 cells (Fig. S2G). Subsequently, we obtained Th17 cells by inducing the differentiation of circulating human CD4^+^ T cells. The concentrations of IL‐17A in Th17 conditioned media were measured by ELISAs (Fig. S3A). The conditioned culture media from Th17 cells were collected and individually added to the HCCLM6 and HCC1664 cell media for 2 weeks to test their effect on EMT of HCC cells. As shown, HCCLM6 cells and HCC1664 cells were induced into a mesenchymal phenotype (Fig. 2E). Consistently, the conditioned culture media of Th17 cells also induced the loss of E‐cadherin and ZO‐1 expression from the cell membrane and increased N‐cadherin and vimentin in HCCLM6 cells and HCC1664 cells (Figs 2F–I and S3B). Moreover, an IL‐17‐neutralizing mAb partially upregulated E‐cadherin and ZO‐1 expression and downregulated N‐cadherin and vimentin in HCC cells stimulated with the conditioned culture media of Th17 cells (Fig. S3C). Furthermore, EMT markers were clearly changed as early as 24 h after treatment with culture media from Th17 cells and exogenous IL‐17A (Figs 2J and S3D). Collectively, these data suggest that IL‐17A induces the EMT phenotype in HCC cells to promote metastasis.

### 3.3 IL‐17A promotes the colonization of HCC cells

**Figure 3:**
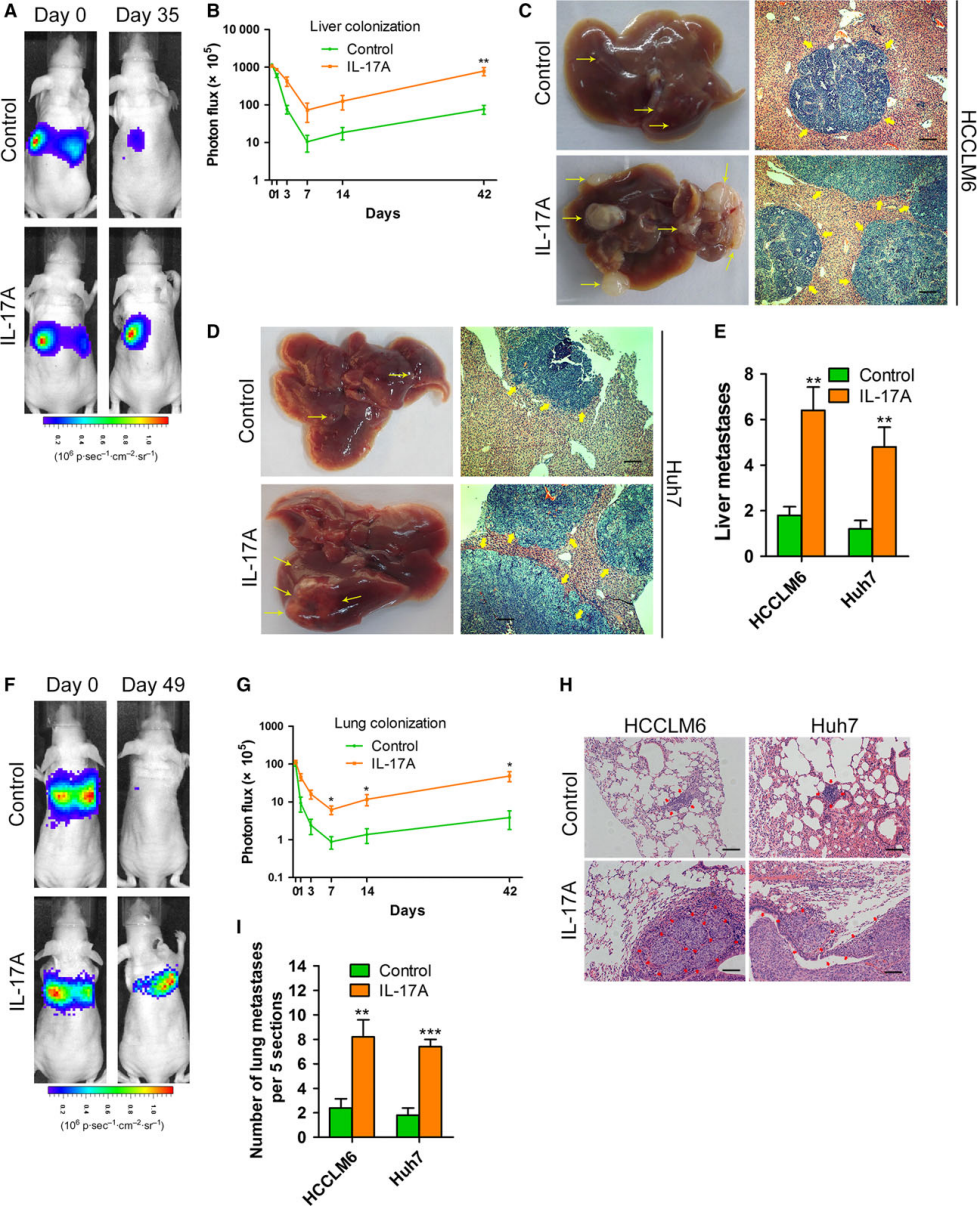
The roles of IL‐17A in metastatic colonization of HCC cells *in vivo*. (A–E) HCCLM6 or Huh7 cells were inoculated intrasplenically into nude mice. Mice (*n* = 5/group) were treated with IL‐17A (1 μg per day, intraperitoneally) or PBS (control) for 6 weeks. (A) Representative photographs of mice after intrasplenic injection with HCCLM6 cells at the indicated times by imaging with the IVIS Imaging System. (B) Luciferase signal intensities of mice in each group over time after intrasplenic injection with HCCLM6 cells. (C,D) Representative views of intrahepatic metastasis foci of HCCLM6 (C) or Huh7 (D) cells in the liver metastasis model. Yellow arrows: metastatic foci. Scale bars = 200 μm. (E) The number of liver metastases in mice from (C) and (D). (F–I) The lung metastasis model was established by injecting HCCLM6 or Huh7 cells directly into the tail veins of nude mice. Mice (*n* = 5/group) were treated with IL‐17A or PBS for 6 weeks. (F) Representative images of mice after tail vein injection with HCCLM6 cells at the indicated times by imaging with the IVIS Imaging System. (G) Luciferase signal intensities of mice over time after tail vein injection with HCCLM6 cells. (H) Haematoxylin/eosin‐stained images of lung tissues isolated from mice as indicated. Scale bars = 200 μm. (I) The number of metastatic nodules in the lungs from mice as indicated. All data are shown as mean ± SD. *: *P* < 0.05, **: *P* < 0.01, ***: *P* < 0.001, compared with the control group.

To explore the influence of IL‐17A on the late step of metastatic colonization in the liver, we inoculated HCCLM6 and Huh7 cells intrasplenically into nude mice to establish a liver metastasis model. Because HCCLM6 cells expressed firefly luciferase, the process of lung metastasis was dynamically monitored using an *in vivo* imaging system. The photon flux images and curves indicated that intraperitoneal injection of IL‐17A promotes liver colonization of HCCLM6 cells (Fig. 3A,B). After 6 weeks, the mice were sacrificed. The number of intrahepatic metastasis foci in the IL‐17A‐injected group was significantly increased compared with that of the control group (Fig. 3C–E). Next, we further investigated the effects of IL‐17A on lung colonization by injecting HCCLM6 and Huh7 cells directly into the tail veins of nude mice. The *in vivo* imaging system and counting of pulmonary metastasis foci suggested an increase in the lung metastasis burden generated by IL‐17A injection (Fig. 3F–I). Together, these results demonstrate that IL‐17A promotes the liver and lung colonization of HCC cells.

### 3.4 The pro‐EMT and pro‐colonization role of IL‐17A requires the activation of AKT

**Figure 4:**
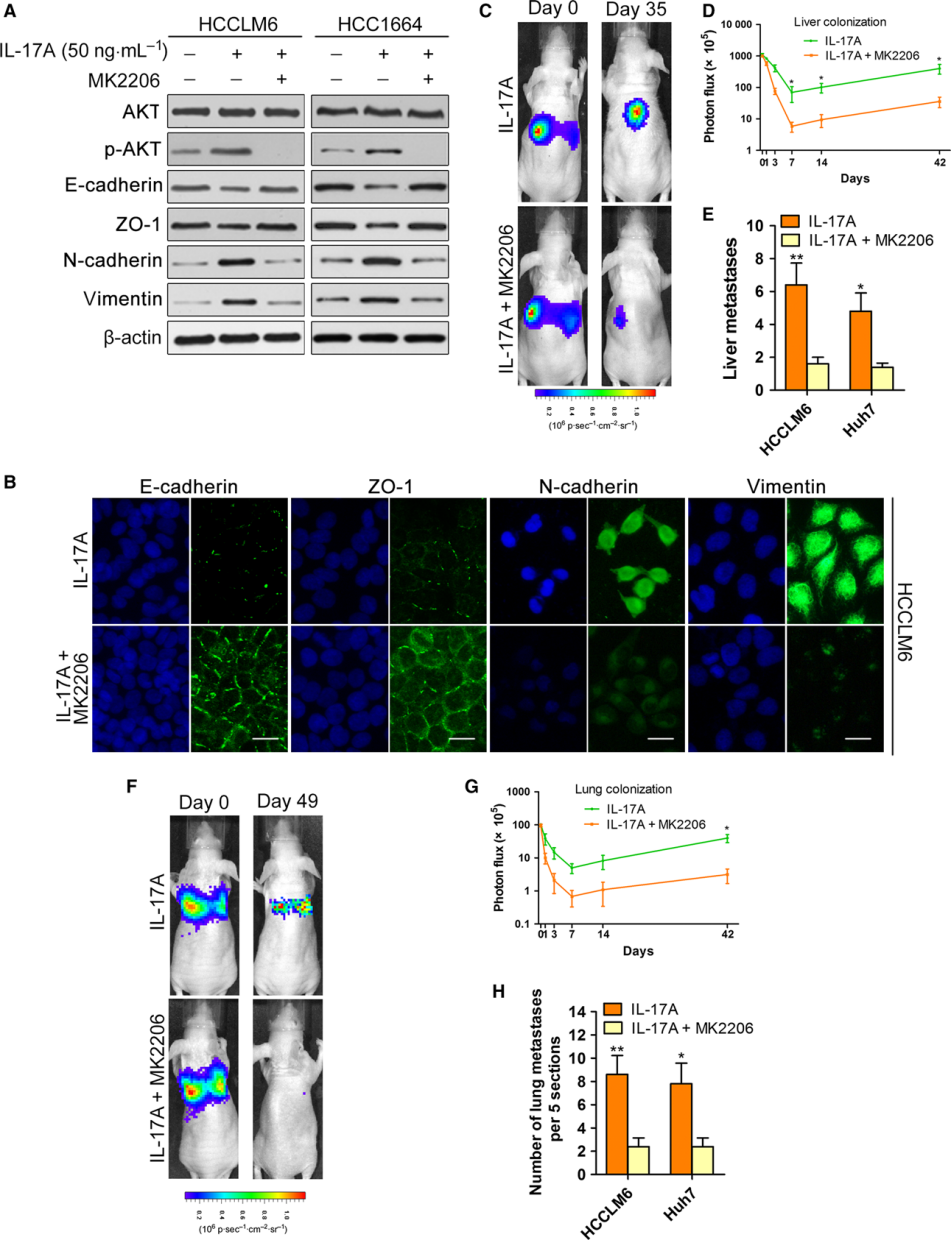
The pro‐EMT and pro‐colonization role of IL‐17A depends on the activation of AKT. (A) The indicated cell lines were exposed to PBS or IL‐17A (50 ng·mL^−1^) or IL‐17A + MK2206 (5 μm) as indicated for 72 h and analysed by western blotting with the indicated antibodies. (B) Immunofluorescence microscopy analysis of the expression of EMT markers in HCCLM6 cells treated with IL‐17A or IL‐17A + MK2206. Scale bars = 50 μm. (C–E) HCCLM6 or Huh7 cells were inoculated intrasplenically into nude mice. Mice (*n* = 5/group) were treated with IL‐17A (1 μg·mouse^−1^ per day, intraperitoneally) or IL‐17A + MK2206 (60 mg·kg^−1^ per 2 days, orally). (C) Representative images of mice after intrasplenic injection with HCCLM6 cells. (D) Luciferase signal intensities of mice in each group over time after intrasplenic injection with HCCLM6 cells. (E) The number of liver metastases in mice. (F‐H) The lung metastasis model was established by injecting HCCLM6 or Huh7 cells directly into the tail veins of nude mice. Mice (*n* = 5/group) were treated as indicated. (F) Representative images of mice after tail vein injection with HCCLM6 cells. (G) Luciferase signal intensities of mice over time after tail vein injection with HCCLM6 cells. (H) The number of metastatic nodules in the lungs from mice as indicated. All data are shown as mean ± SD. *: *P* < 0.05, **: *P* < 0.01.

Our previous study indicated that IL‐17A activated AKT to promote tumour progression (Gu *et al*., 2011); therefore, we investigated whether AKT phosphorylation mediated the effects of IL‐17A on EMT and colonization. We treated IL‐17A‐stimulated HCCLM6 and HCC1664 cells with MK2206, a highly potent and selective inhibitor of AKT (Hirai *et al*., 2010). MK2206 (5 μm) effectively inhibited the phosphorylation of AKT (Fig. 4A) and caused mesenchymal HCC cells induced by IL‐17A to revert to an epithelial phenotype (Fig. S4A). Furthermore, the decreased E‐cadherin and ZO‐1, as well as the increased N‐cadherin and vimentin in HCCLM6 and HCC1664 cells induced by IL‐17A, were completely abrogated by MK2206 (Figs 4A,B and S4B–D). Then, we analysed the role of AKT activation on IL‐17A‐stimulated cell colonization *in vivo*. In a liver metastasis model established by inoculating HCCLM6 and Huh7 cells intrasplenically into nude mice, MK2206 abolished the liver colonization‐promoting role of IL‐17A based on *in vivo* bioimaging and gross observation of liver metastasis foci (Fig. 4C–E). Consistently, MK2206 significantly reduced the IL‐17A‐stimulated lung metastasis burden of HCCLM6 and Huh7 cells in a lung metastasis model in which cells were injected directly into the tail veins of nude mice (Fig. 4F–H). Collectively, the results suggest that the activation of AKT is responsible for the promoting effects of IL‐17A on EMT and colonization.

### 3.5 IL‐6 is involved in the regulation of IL‐17A on EMT and colonization

**Figure 5:**
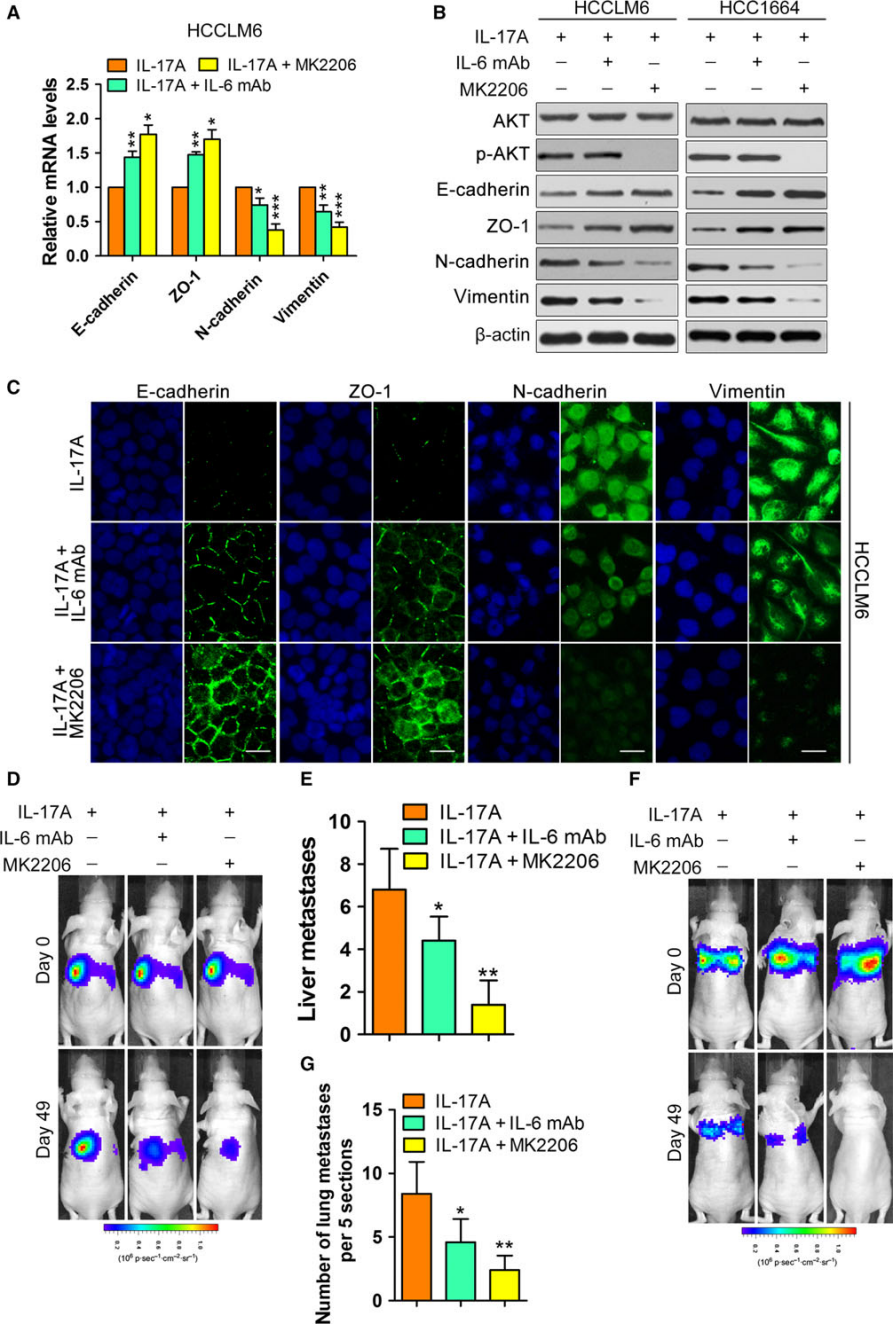
Effects of IL‐6 on pro‐EMT and pro‐colonization induced by IL‐17A. (A–C) The indicated cell lines were exposed to IL‐17A (50 ng·mL^−1^) or IL‐17A + IL‐6 mAb (10 ng·mL^−1^) or IL‐17A + MK2206 (5 μm) for 72 h. (A) The mRNA levels of EMT markers in the indicated cells. (B and C) The protein levels of EMT markers in the indicated cells measured by western blotting (B) and immunofluorescence (C). Scale bars = 50 μm. (D,E) HCCLM6 were inoculated intrasplenically into nude mice. Mice (*n* = 5/group) were treated with IL‐17A (1 μg·mouse^−1^ per day, intraperitoneally) or IL‐17A + IL‐6 mAb (1 mg·mouse^−1^ per week, intraperitoneally) or IL‐17A + MK2206 (60 mg·kg^−1^ per 2 days, orally). (D) Representative images of mice. (E) The number of liver metastases in mice. (F–G) The lung metastasis model was established by injecting HCCLM6 cells directly into the tail veins of nude mice. Mice (*n* = 5/group) were treated as indicated. (F) Representative images of mice. (G) The number of metastatic nodules in the lungs from mice as indicated. All data are shown as mean ± SD. *: *P* < 0.05, **: *P* < 0.01, ***: *P* < 0.001.

Previous studies showed that the secretion of IL‐6 was increased by IL‐17A‐mediated AKT activation and associated with HCC development (Gu *et al*., 2011). Thus, we first detected the expression of IL‐6 mRNA before and after stimulation with IL‐17A and determined whether the presence of IL‐6 in conditioned media is dependent on AKT activation. The results showed that the expression of IL‐6 mRNA was increased after stimulation with IL‐17A and that IL‐17A‐induced IL‐6 secretion was dependent on AKT activation (Fig. S5A,B). Then, we further tested the contribution of IL‐6 to the pro‐EMT and pro‐colonization role of IL‐17A. An IL‐6‐neutralizing mAb partially upregulated E‐cadherin and ZO‐1 expression and downregulated N‐cadherin and vimentin of HCC cells stimulated with IL‐17A (Figs 5A–C and S5C,D). However, compared with those of HCC cells stimulated by IL‐17A and MK2206, the HCC cells treated with IL‐17A and IL‐6 mAb had low expression levels of E‐cadherin and ZO‐1 and high expression levels of N‐cadherin and vimentin. Therefore, unlike MK2206, the IL‐6 mAb did not completely abolish IL‐17A‐induced EMT in HCCLM6 and HCC1664 cells. Additionally, IL‐6 mAb partially attenuated the liver and lung metastasises burden of HCCLM6 and Huh7 cells stimulated by IL‐17A (Figs 5D–G and S5E,F). The adverse effect of MK2206 on the pro‐colonization role of IL‐17A was more obvious than that of IL‐6 mAb. Taken together, IL‐17A‐induced IL‐6 as a downstream target of activated AKT partially promotes EMT and the colonization of HCC cells. Moreover, the pro‐EMT and pro‐colonization roles of IL‐17A are dependent not only on upregulated IL‐6 production but also on other pathways activated by AKT.

### 3.6 Secukinumab/sorafenib combination was more efficacious than sorafenib monotherapy in inhibiting HCC growth and metastasis *in vivo*

**Figure 6:**
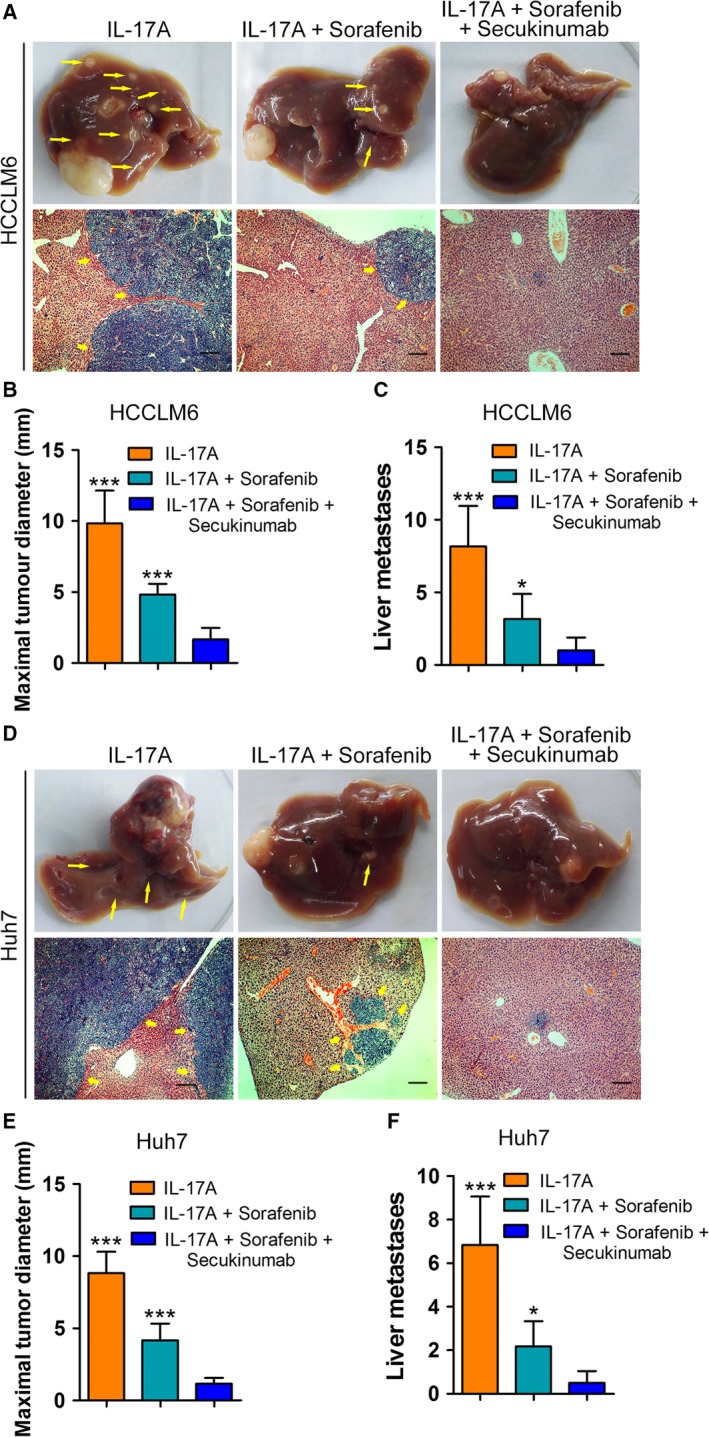
Combined therapy using both secukinumab and sorafenib shows better inhibition of tumour growth and metastasis than that of sorafenib monotherapy. The orthotopic implant models were established using HCCLM6 and Huh7 cells. The orthotopically implanted tumours were allowed to grow for 2 weeks. Mice were then randomized into three groups (*n* = 6/group): vehicle control (PBS, orally), sorafenib (30 mg·kg^−1^ per day, orally) or sorafenib + secukinumab (500 μg·mouse^−1^ per week, intraperitoneally), for 5 consecutive weeks. (A,D) Representative livers and views of intrahepatic metastasis foci of HCCLM6 (A) or Huh7 (D) cells in the orthotopically implanted mice Yellow arrows: metastatic foci. Scale bars = 200 μm. (B,E) Maximal tumour diameters in livers of orthotopically implanted mice. (C,F) Number of liver metastases in mice. All data are shown as mean ± SD. *: *P* < 0.05, ***: *P* < 0.001.

To assess the efficacy of IL‐17A targeting therapeutics in preventing HCC progression, we administered secukinumab (Langley *et al*., 2014), an anti‐human IL‐17A monoclonal antibody approved for several inflammatory disorders, to treat an orthotopic implantation tumour model in combination with sorafenib. We used subcutaneous tumour tissues formed by HCCLM6 and Huh7 cells to establish orthotopically implanted models. The orthotopically implanted tumours were allowed to grow for 2 weeks. Then, the models were randomly divided into the IL‐17A group, IL‐17A + sorafenib group, and IL‐17A + secukinumab + sorafenib combination group. Treatment was initiated on day 15. After 5 weeks, sorafenib inhibited tumour growth and intrahepatic metastases, whereas the combined treatment ofwith secukinumab and sorafenib was significantly more efficacious than sorafenib monotherapy (Fig. 6A–F). Promisingly, targeting the IL‐17A pathway may be a future treatment option for HCC management, and secukinumab may be a potential drug candidate in the clinic.

### 3.7 The combination of E‐cadherin expression and the percentage of IL‐17A‐positive cells serves as a prognostic factor for patients with HCC at an early stage after hepatectomy

**Figure 7:**
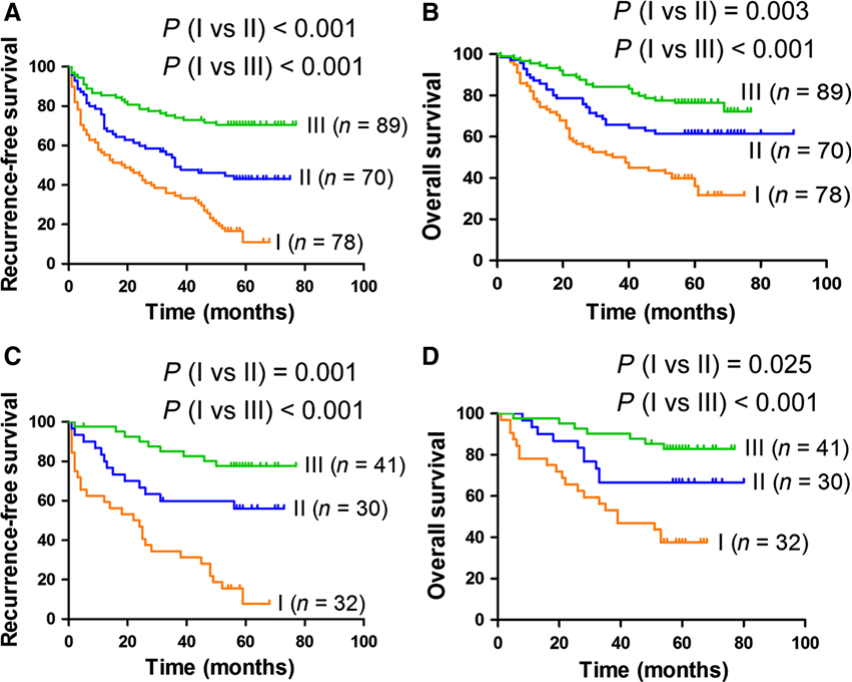
Prognostic value of E‐cadherin expression and the percentage of IL‐17A‐positive cells for patients with HCC at an early stage after hepatectomy. Patients with HCC at an early stage from the TMA were used for the prognostic analysis. Patients were classified into three groups: group I, high percentage of IL‐17A+ cells and low expression of E‐cadherin; group II, high percentage of IL‐17A+ cells and high expression of E‐cadherin, or low percentage of IL‐17A+ cells and low expression of E‐cadherin; and group III, low percentage of IL‐17A+ cells and high expression of E‐cadherin. (A,B) The RFS (A) and OS (B) curves of patients at BCLC stage 0‐A (*n* = 237). (C,D) The RFS (C) and OS (D) curves of patients at TNM stage I (*n* = 103).

Tumour staging systems based on multiple clinicopathological characteristics [e.g. Barcelona clinic liver cancer (BCLC) and TNM staging systems] can be used to predict outcomes of patients with HCC. However, an interaction between the tumour stage and actual prognosis is not always observed. Some patients with early‐stage HCC show a poor prognosis, presenting clinicians with a major challenge in prognostic prediction for these patients. Therefore, we further investigated the prognostic value of IL‐17A and E‐cadherin in combination in the TMA containing 313 patients with HCC. Patients at BCLC stage 0‐A (*n* = 237) or TNM stage I (*n* = 103) were included. Patients were classified into three groups: group I, high expression of IL‐17A and low expression of E‐cadherin; group II, high expression of both markers or low expression of both markers; and group III, low expression of IL‐17A and high expression of E‐cadherin. A univariate analysis revealed that significant differences in recurrence‐free survival (RFS) and overall survival (OS) were detected among the three groups (Fig. 7A–D). A Cox proportional hazards model showed that compared with group III, the hazard ratios of group I were 0.453 (95% CI, 0.359–0.571; *P* < 0.001) for RFS and 0.523 (95% CI, 0.404–0.677; *P* < 0.001) for OS of patients at BCLC stage 0‐A (Table S1). These data support the hypothesis that high expression of IL‐17A and low expression of E‐cadherin predict the outcome of patients with HCC at an early stage.

## 4 Discussion

As a crucial pro‐inflammatory cytokine, IL‐17A promotes HCC progression and is associated with HCC prognosis (Gu *et al*., 2011; Liao *et al*., 2013; Wu *et al*., 2012a; Zhang *et al*., 2009). However, the role and mechanisms of IL‐17A on the invasion–metastasis cascade of HCC remain largely unknown. In this study, we report that IL‐17A induces EMT and promotes HCC cell colonization at the site of metastasis by activating AKT signalling. We also provide a rationale to preclinically evaluate the effects of anti‐IL‐17A mAb on blocking HCC metastasis. Furthermore, the combination of IL‐17A and E‐cadherin predicts the outcome of patients with HCC at an early stage.

In the orthotopic xenograft model in nude mice, we found that IL‐17A promoted intrahepatic and pulmonary metastases of HCC cells. Moreover, the number of CTCs in the mouse circulation was increased by IL‐17A stimulation, indicating that more HCC cells successfully escape from the primary tumour site and struggle to survive in the bloodstream due to IL‐17A stimulation. Additionally, detection of the IL‐17A+ cell percentage in clinical samples also supported the hypothesis that IL‐17A plays a pro‐metastatic role in HCC, consistent with the effects on diverse cancer types (McAllister and Kolls, 2015; Wu *et al*., 2016; Zhu *et al*., 2008). Metastasis is a complex multistep process that involves early invasion and late colonization of cancer cells (Tao *et al*., 2013). Thus, we further explored the role of IL‐17A in different steps of metastasis. EMT plays a crucial role in the early steps of metastasis by endowing cells with a more motile, invasive potential (Thiery *et al*., 2009). Our data indicated that the exogenous and Th17‐produced IL‐17A induced the EMT of HCC cells. A recent report suggested that IL‐17A promotes prostate cancer via MMP7‐induced EMT (Zhang *et al*., 2017), which further supports the regulation of EMT by IL‐17A. Li *et al*. (2011) reported that the recombinant human IL‐17A could not promote the EMT in PLC8024 (a HCC cell line) cells. However, the study did not detect the expression of IL‐17A receptor in PLC8024 cells. Thus, the inconsistent results may be due to the low or absent expression of the IL‐17A receptor in the cells. Although there is significant and continuous cancer cell intravasation into the circulation, only a small minority of these cells form colonies at a secondary site, initiating cell division to form metastatic focus (Chambers *et al*., 2002). Hence, colonization is a rather inefficient process and has a critical effect on the ultimate metastasis. In our intrasplenic inoculation and tail vein injection xenograft models, we found that IL‐17A promoted the colonization of HCC cells. Therefore, IL‐17A accelerates the invasion–metastasis cascade of HCC by inducing early EMT and promoting late colonization.

Our previous study indicated that IL‐17A significantly induced AKT activation in HCC cells, which was also validated in this study. Recently, a study also suggested that IL‐17A activates AKT signalling in B‐cell acute lymphoblastic leukaemia (Bi *et al*., 2016). We thus hypothesize that the activation of AKT mediates the IL‐17A‐induced invasion–metastasis cascade of HCC. MK2206, an inhibitor of AKT, inhibited HCC cellular proliferation via induction of apoptosis and cell cycle arrest when used alone (Wilson *et al*., 2014). When combined with IL‐17A, MK2206 completely reverted the IL‐17A‐induced EMT *in vitro* and abolished the colonization‐promoting role of IL‐17A *in vivo*. Previous studies indicated that activation of the AKT pathway downregulates E‐cadherin expression and induces EMT via the upregulated expression of snail (Grille *et al*., 2003; Larue and Bellacosa, 2005), and the AKT pathway is involved in the metastatic colonization of tumour cells (Wu *et al*., 2012b). This also supports regulation of the AKT pathway on EMT and colonization. IL‐17A elicits the secretion of diverse inflammatory mediators in HCC cells, particularly IL‐6 (Gu *et al*., 2011). IL‐6 plays a vital role in bridging chronic inflammation to HCC progression and is correlated with tumour metastasis (Chang *et al*., 2015; Kao *et al*., 2015). Furthermore, an IL‐6‐neutralizing antibody diminished the invasion‐promoting effect of mesenchymal stem cell conditioned medium‐treated HCC cells (Mi and Gong, 2017). In this study, we found that IL‐17A‐induced IL‐6 as a downstream target of activated AKT partially promotes EMT and the colonization of HCC cells, whereas upregulated IL‐6 production is not the only factor for the pro‐metastasis role of IL‐17A. Notably, we could not exclude the impact of MK2006 itself on HCC cell *in* *vivo* assays (Wilson *et al*., 2014). The reduced potential of HCC cells to colonize the target organ observed in the presence of MK2206 and IL‐17A could be a consequence of the effect of MK2006 itself on the cell cycle or cell viability and not due to its ability to block signalling cascades activated downstream of IL‐17A.

Although sorafenib is currently the only first‐line systemic therapy approved for the treatment of advanced HCC (Llovet *et al*., 2008), the objective response rate to sorafenib treatment is modest and unsatisfactory (Cheng *et al*., 2009; Gauthier and Ho, 2013). It was reported that sorafenib resistance is mediated by EMT and associated with the serum IL‐17A level in patients with HCC (Cho *et al*., 2017; Mir *et al*., 2017). Therefore, blocking the IL‐17A pathway may increase the efficacy of sorafenib. Secukinumab, an IL‐17A‐neutralizing antibody, is approved for a variety of inflammatory diseases (Langley *et al*., 2014) and has shown efficacy for multiple myeloma treatment in a preclinical study (Prabhala *et al*., 2016). Remarkably, we found that combined therapy using both secukinumab and sorafenib exhibited better inhibition of tumour growth and metastasis than that of sorafenib monotherapy, suggesting that secukinumab can be used as an adjuvant to enhance the clinical response to sorafenib, resulting in a longer duration of responses. An efficacy trial of secukinumab and sorafenib was conducted with exogenous IL‐17A. This would be similar to the clinical scenario if the mice are exposed to endogenous IL‐17A.

The BCLC and the TNM staging systems are the most frequently used systems to stage patients with HCC and have prognostic functions (Bruix *et al*., 2016). Based on the staging systems, patients with early‐stage tumours are identified with a lower risk for recurrence. However, some early‐stage patients may continue to show a poor prognosis after hepatectomy. Therefore, predicting the outcome of these patients is a major challenge for attending clinicians. Here, we demonstrated the prognostic value of IL‐17A/E‐cadherin combination for patients with HCC at an early stage. For early‐stage patients with a high risk of recurrence, close follow‐up and appropriate adjuvant therapies should be recommended to prolong survival.

In summary, our research demonstrated that IL‐17A promotes the invasion–metastasis cascade by activating the AKT pathway and provides a rationale for the combination of secukinumab and sorafenib in advanced HCC treatment. The findings of this study have significant implications regarding our understanding of HCC metastasis pathogenesis. These new insights may facilitate the development of combination therapy in the future to impact the survival of patients with advanced HCC.

## 5 Conclusions

Overall, our findings of the pro‐metastasis effects of IL‐17A in HCC, including the induction of early EMT and promotion of late colonization, depend on activation of the AKT pathway. Secukinumab is a promising candidate for further development as a combination therapy with sorafenib for human HCC treatment.

## Authors’ contributions

FG, WZ and FY designed the study and supervised the experiments. QX and JY wrote the manuscript. QX, JY, GH, XG, SY and YY performed most of the experiments and analysed the results. YY, HL and ZP collected clinical samples and supervised clinicopathological data.

## Ethics approval and consent to participate

All procedures performed involving human participants were in accordance with the ethical standards of the institutional and national research committee and with the 1964 Helsinki Declaration and its later amendments or comparable ethical standards. Informed consent was obtained from each patient, and this study was approved by the Eastern Hepatobiliary Surgery Hospital Research Ethics Committee.

## Consent for publication

Not applicable.

## Availability of data and material

The data sets in and/or analysed during the current study are available from the corresponding author on reasonable request.

## Supporting information


**Fig. S1**. The percentage of IL‐17A+ cells is relevant to the expression of EMT markers.

**Fig. S2**. The effects of exogenous IL‐17A on EMT of HCC cells *in vitro*.

**Fig. S3**. The effects of Th17 conditioned media on EMT of HCC cells *in vitro*.

**Fig. S4**. IL‐17A requires activation of AKT to promote EMT in HCC cells.

**Fig. S5**. The effects of IL‐6 on pro‐EMT and pro‐colonization induced by IL‐17A.

**Table S1**. Multivariate analysis of several variables for OS and RFS of patients at BCLC stage 0‐A.
**Table S2**. The list of primers used in study.

**Appendix S1**. Material and methods.
