# Supplementary figures and images for: IL‐17A promotes the invasion–metastasis cascade via the AKT pathway in hepatocellular carcinoma
Source: Mol Oncol. 2018 Apr 26;12(6):936–52. doi: 10.1002/1878-0261.12306 (PMC5983223; doi:10.1002/1878-0261.12306)

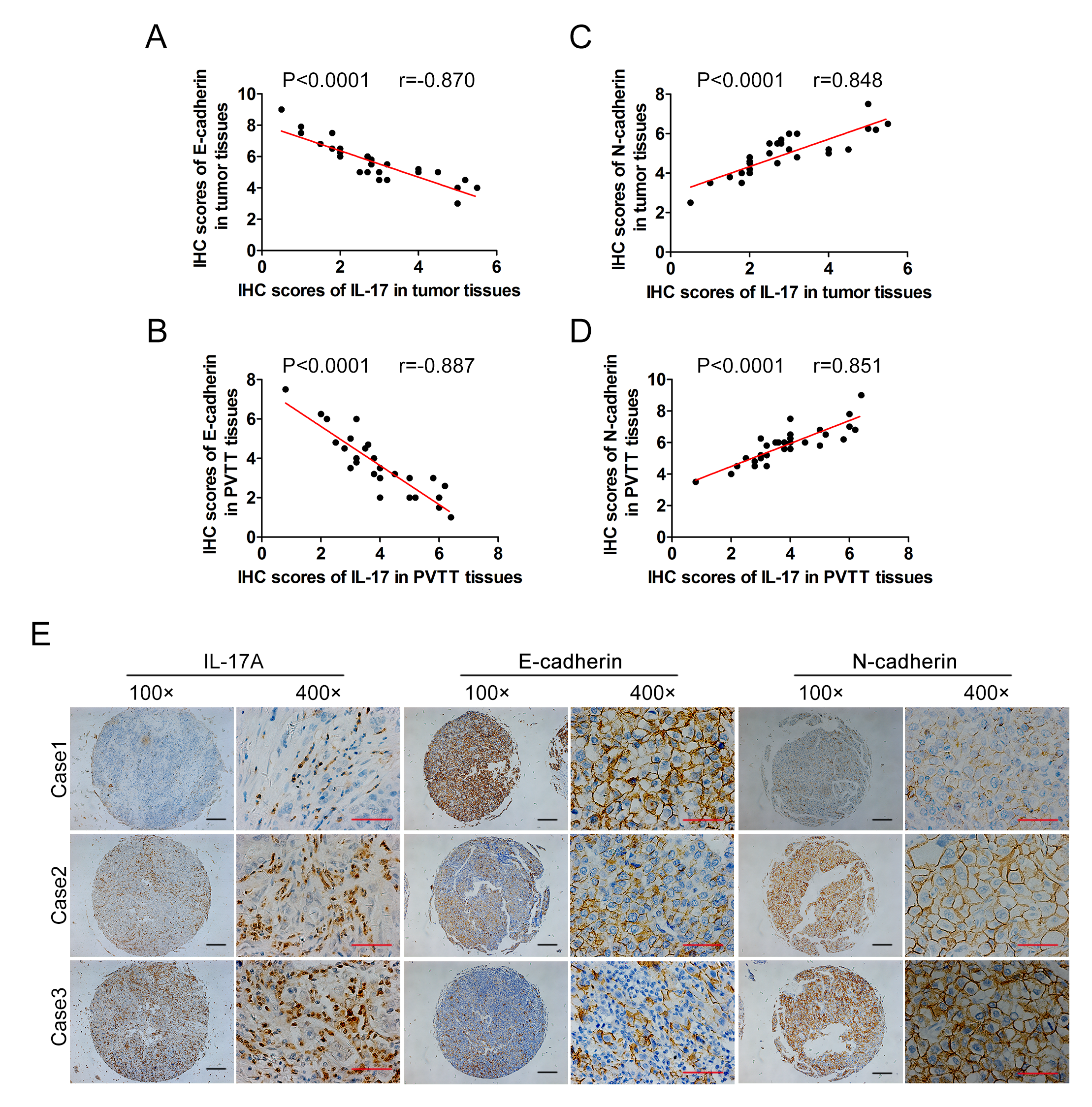

Supplement: Supplementary file 1 — Fig. S1. The percentage of IL‐17A+ cells is relevant to the expression of EMT markers. [file MOL2-12-936-s001.tif]

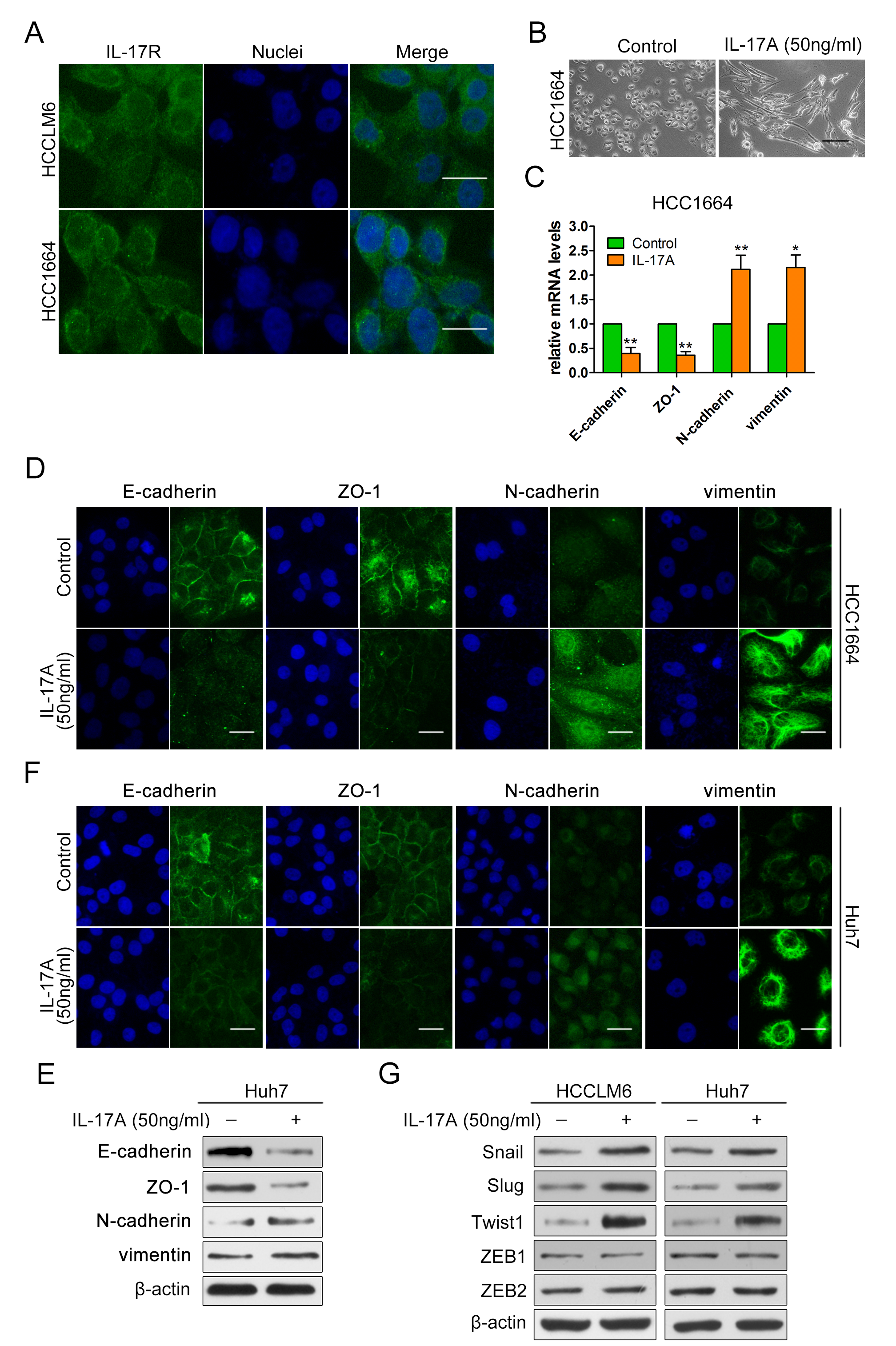

Supplement: Supplementary file 2 — Fig. S2. The effects of exogenous IL‐17A on EMT of HCC cells in vitro. [file MOL2-12-936-s002.tif]

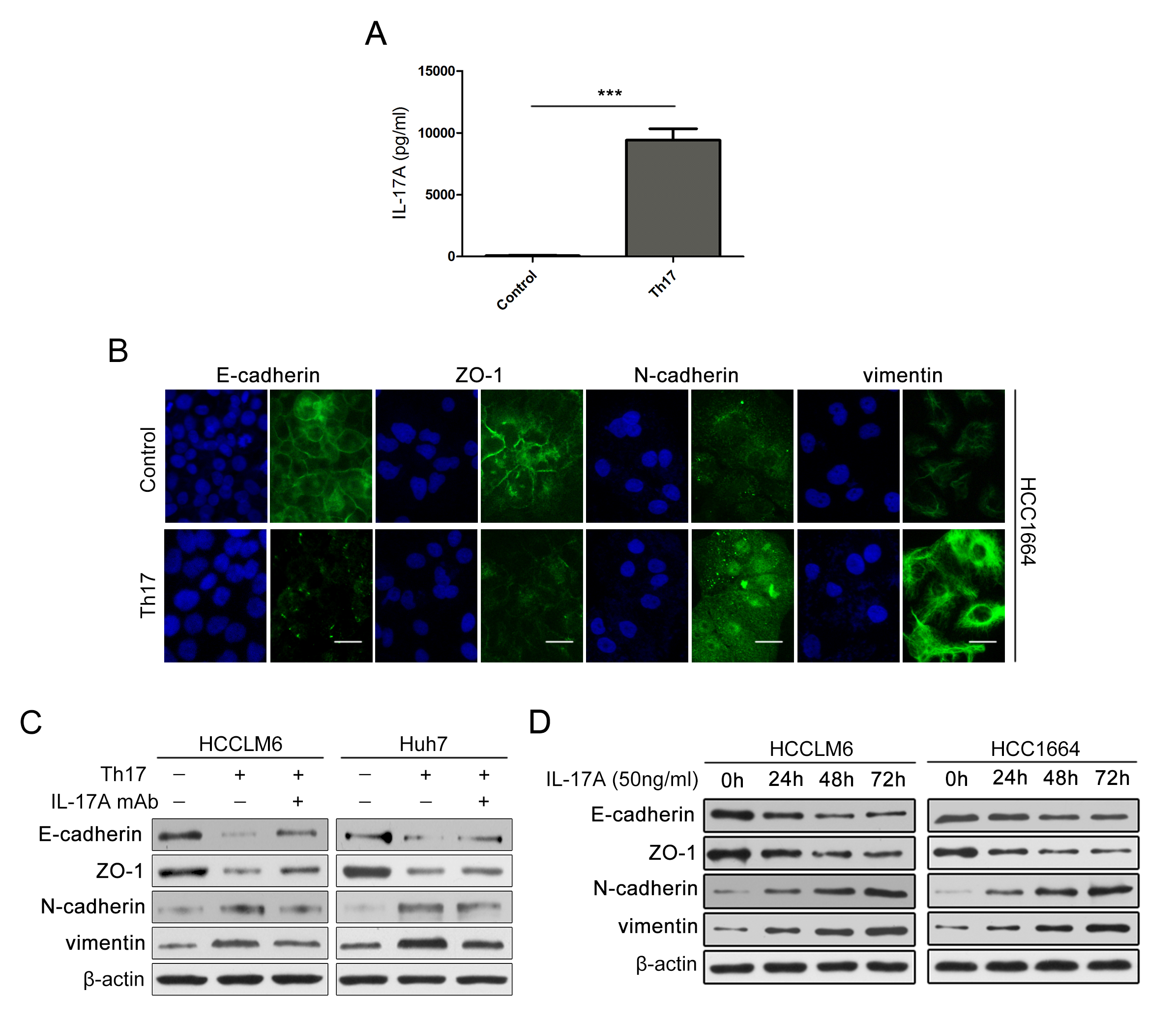

Supplement: Supplementary file 3 — Fig. S3. The effects of Th17 conditioned media on EMT of HCC cells in vitro. [file MOL2-12-936-s003.tif]

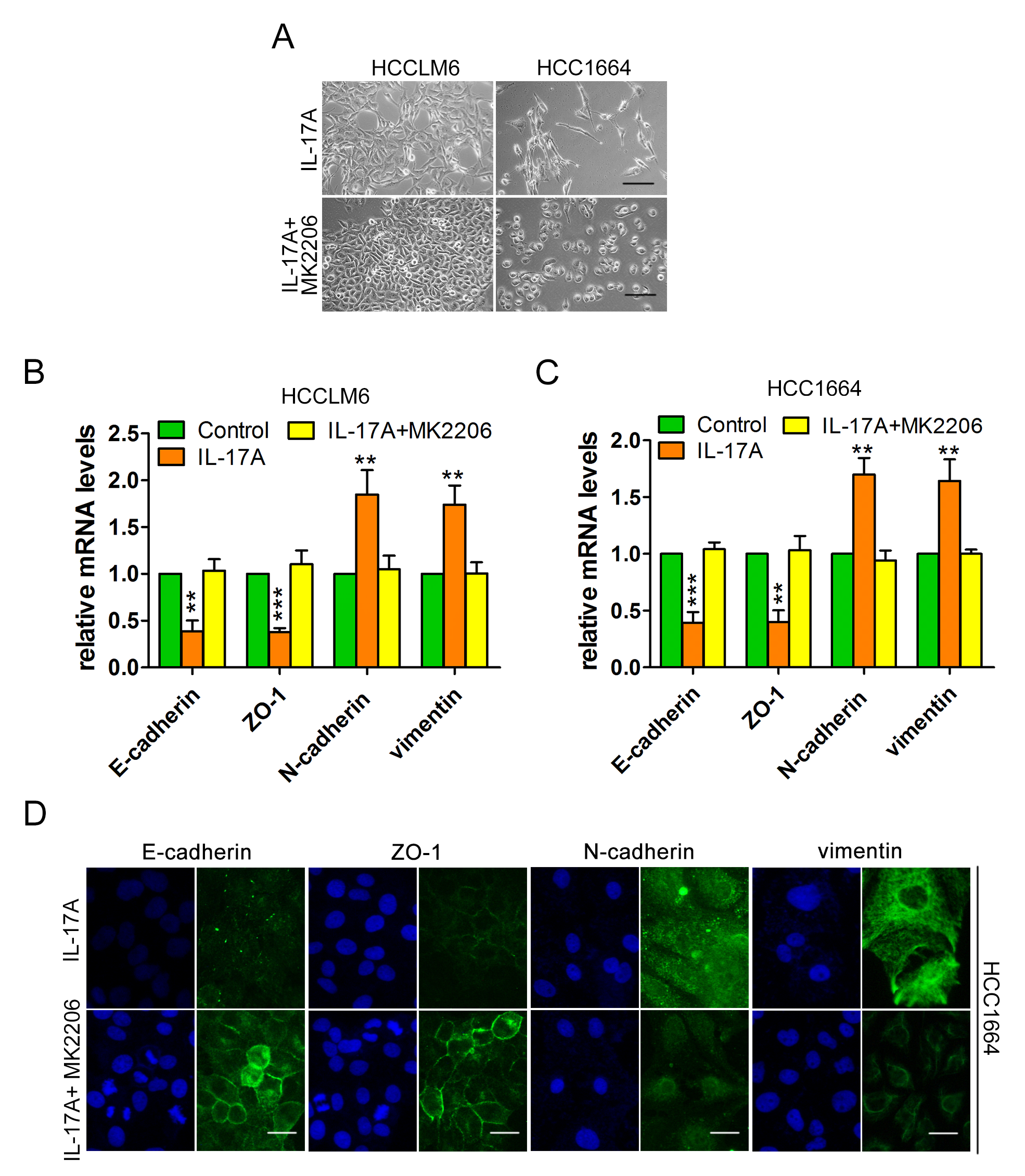

Supplement: Supplementary file 4 — Fig. S4. IL‐17A requires activation of AKT to promote EMT in HCC cells. [file MOL2-12-936-s004.tif]

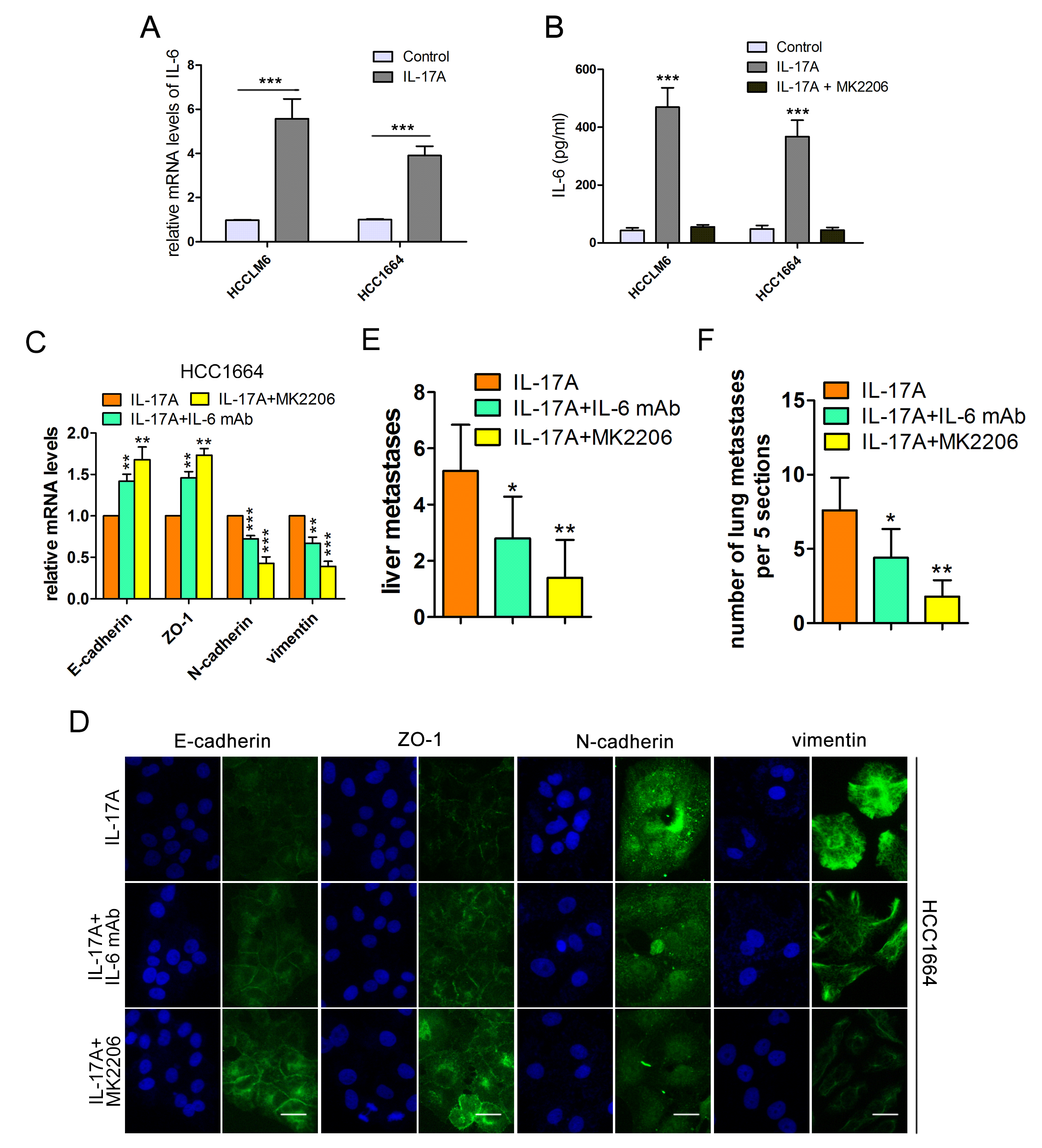

Supplement: Supplementary file 5 — Fig. S5. The effects of IL‐6 on pro‐EMT and pro‐colonization induced by IL‐17A. [file MOL2-12-936-s005.tif]
