# Supplementary material for: IL‐17A promotes the invasion–metastasis cascade via the AKT pathway in hepatocellular carcinoma
Source: Mol Oncol. 2018 Apr 26;12(6):936–52. doi: 10.1002/1878-0261.12306 (PMC5983223; doi:10.1002/1878-0261.12306)
Supplement: Supplementary file 6 — Table S1. Multivariate analysis of several variables for OS and RFS of patients at BCLC stage 0‐A. Table S2. The list of primers used in study. [file MOL2-12-936-s006.docx]

| Variable^▲^ | Recurrence-Free Survival (n=167) | |  | Overall Survival (n=167) | |
| --- | --- | --- | --- | --- | --- |
|  | Hazardratio(95 % CI) | P-value |  | Hazardratio(95 % CI) | P-value |
| HBe antigen, positive | - | N.S. |  | - | N.S. |
| a-Fetoprotein, > 20 ng/mL | - | N.S. |  | - | - |
| Liver cirrhosis, Present | 1.324(1.023–1.823) | 0.031^★^ |  | 1.777(1.023-3.088) | 0.041^★^ |
| Tumor size(cm), >5 | - | N.S. |  | 2.099(1.301-3.385) | 0.002^★^ |
| Differentiation, III + IV | - | N.S. |  | - | N.S. |
| Micro-vascular invasion, Present | - | N.S. |  | - | - |
| Encapsulation, Absent/Incomple | - | N.S. |  | - | - |
| Combination of IL-17A and E-cadherin, Group I vs III | 0.453(0.359-0.571) | <0.001^★^ |  | 0.523(0.404-0.677) | <0.001^★^ |

Supplementary Table 1 Multivariate analysis of several variables for OS and RFS of patients at BCLC stage 0-A

▲Variables were adopted for their prognostic significance by univariate analysis.

★P<0.05 by Cox proportional hazards regression model.

N.S., not significant.

Supplementary Table 2.The list of primers used in study.

| Name | Sequence |
| --- | --- |
| E-cadherin | Forward: GCCCCATCAGGCCTCCGTTT |
|  | Reverse: ACCTTGCCTTCTTTGTCTTTGTTGGA |
| ZO-1 | Forward: CACGCAGTTACGAGCAAG |
|  | Reverse: TGAAGGTATCAGCGGAGG |
| N-cadherin | Forward: TGGACCATCACTCGGCTTA |
|  | Reverse: ACACTGGCAAACCTTCACG |
| vimentin | Forward: CCTGAACCTGAGGGAAACTAA |
|  | Reverse: GCAGAAAGGCACTTGAAAGC |
| IL-6 | Forward: AAATTCGGTACATCCTCGACGG |
|  | Reverse: GGAAGGTTCAGGTTGTTTTCTGC |
| β-actin | Forward: GGGAAATCGTGCGTGACATTAAG |
|  | Reverse: TGTGTTGGCGTACAGGTCTTTG |
