# Supplementary material for: IL‐17A promotes the invasion–metastasis cascade via the AKT pathway in hepatocellular carcinoma
Source: Mol Oncol. 2018 Apr 26;12(6):936–52. doi: 10.1002/1878-0261.12306 (PMC5983223; doi:10.1002/1878-0261.12306)
Supplement: Supplementary file 7 — Appendix S1. Material and methods. [file MOL2-12-936-s007.docx]

**Supplementary figure legends**

**Figure S1, related to Figure 1**

The percentage of IL-17A+ cells is relevant to the expression of EMT markers.

(A&B) The correlation between IL-17A+ cells percentage and E-cadherin level in human HCC tissues (A) (n=30) and matched PVTT tissues (B) (n=30). (C&D) The correlation between IL-17A+ cells percentage and N-cadherin level in human HCC tissues (C) (n=30) and matched PVTT tissues (D) (n=30). (E) Representative immunohistochemistry stains of IL-17A, E-cadherin and N-cadherin in TMA. Black scale bars = 100 µm. Red scale bars = 50 µm.

**Figure S2, related to Figure 2**

The effects of exogenous IL-17A on EMT of HCC cells *in vitro*.

(A) Expression of the IL-17A receptor was assessed by immunofluorescence staining and confocal microscopy in HCCLM6 and HCC1664 cells. Scale bars = 20 µm. (B-D) HCC1664 cells were stimulated continuously with exogenous IL-17A (50 ng/ml) or PBS (control) for two weeks. (B) Phase-contrast micrographs of indicated HCC1664 cells. Scale bars = 100 µm. (C) The mRNA levels of EMT markers in HCC1664 cells. (D) Immunofluorescence microscopy analysis of the localization and expression of EMT markers in indicated HCC1664 cells. Scale bars = 50 µm. (E) Western blotting showed the protein levels of EMT markers in the indicated Huh7 cells. (F) Immunofluorescence microscopy analysis of the localization and expression of EMT markers in indicated Huh7 cells. Scale bars = 50 µm. (G) Western blotting showed the protein levels of EMT-transcription factors in the indicated HCCLM6 and Huh7 cells.

**Figure S3, related to Figure 2**

The effects of Th17 conditioned media on EMT of HCC cells *in vitro*.

(A) The concentrations of the IL-17A in Th17 conditioned media and control were measured by ELISA. All data were shown as mean ± S.D. ***: p< 0.001, compared with control group. (B) Immunofluorescence microscopy analysis of the expression of EMT markers in HCC1664 cells after stimulation with culture solution from Th17 cells for two weeks. Scale bars = 50 µm. (C) Western blotting showed the protein levels of EMT markers in the indicated HCC1664 and Huh7 cells. (D) Western blotting showed that EMT markers were changed after treatment with purified IL-17A (50 ng/ml) at different point of time.

**Figure S4, related to Figure 4**

IL-17A requires activation of AKT to promote EMT in HCC cells

HCCLM6 cells or HCC1664 cells were stimulated continuously with exogenous IL-17A (50 ng/ml) or IL-17A+MK2206 (5 μM) for two weeks. (A) MK2206 (5 μM) caused mesenchymal HCC1664 cells induced by IL-17A to revert to an epithelial phenotype. Scale bars = 100 µm. (B & C) The mRNA levels of EMT markers in HCCLM6 cells (B) or HCC1664 (C) cells treated as indicated. (D) Immunofluorescence microscopy analysis of the expression of EMT markers in HCCLM6 cells treated as indicated. Scale bars = 50 µm. All data were shown as mean ± S.D. **: p< 0.01, ***: p< 0.001.

**Figure S5, related to Figure 5**

The effects of IL-6 on pro-EMT and pro-colonization induced by IL-17A

(A) The mRNA levels of IL-6 in HCCLM6 and HCC1664 cells treated as indicated. (B) The concentrations of the IL-6 in conditioned media of HCCLM6 and HCC1664 cells treated as indicated were measured by ELISA. ***: p< 0.001, compared with control group. (C) The mRNA levels of EMT markers in HCC1664 cells treated as indicated. (D) Immunofluorescence microscopy analysis of the expression of EMT markers in HCCLM6 cells treated as indicated. Scale bars = 50 µm. (E) The number of liver metastases in the indicated mice. (F) The number of metastatic nodules in the lungs of mice as indicated. All data were shown as mean ± S.D. *: p< 0.05, **: p< 0.01, ***: p< 0.001, for C and F.

**Supplementary material and methods:**

**Clinical samples**

Preoperative investigations of HCC patients were performed as described previously ([Huang et al., 2014](#_ENREF_1)). The patients with positive HBs antigen were the following: MIH (82.5% (33/40)); MAH (75% (30/40)); PVTT (86.7% (26/30)); and TMA (86.9% (272/313)). The patients with positive HBs antigen and a high preoperative serum HBV DNA level (>1,000 IU/ml) received antiviral treatment. Antiviral treatment was performed as described previously ([Huang et al., 2013](#_ENREF_2)).

The surgical procedures were performed on patients with HCC as described previously ([Yang et al., 2010](#_ENREF_6)). Tissue collection was described previously ([Yang et al., 2010](#_ENREF_6)). The MIH and MAH were collected from January 2011 to December 2013. PVTT tissues were collected from January 2013 to December 2016. The tissues for the TMA were randomly collected from January 2006 to September 2010. All specimens were pathologically confirmed at the Eastern Hepatobiliary Surgery Hospital.

Patients with HCC (MIH, MAH and TMA) received check-ups every 2-3 months during the first 2 years and then every 3 months thereafter. The follow-up of MIH and MAH ended in December 2015. The follow-up of TMA tissues ended in January 2013. The follow-up was performed as described previously ([Tao et al., 2015](#_ENREF_5)). For localized recurrent tumour, repeat liver resection was the treatment of choice. Radiofrequency ablation (RFA), microwave coagulation (MCT) or gamma ray radiotherapy was used in patients with localized tumours who were not candidates for liver resection. For multiple intrahepatic recurrent tumours that were beyond liver resection or local ablative therapy, transcatheter arterial chemoembolization (TACE) was administered.

**Isolation of circulating tumour cells**

Whole blood samples were collected from mice 5 weeks after orthotopic xenografting using HCCLM6 or Huh7 cells labelled with GFP by tail vein bleeding into K3-EDTA-coated tubes. Peripheral blood mononuclear cells (PBMCs, including CTCs) were enriched by Ficoll-Hypaque gradient centrifugation ([Ma et al., 2017](#_ENREF_3)). To exclude the interference of autofluorescence and to select the appropriate gate for flow cytometry, PBMCs from the blood of mice without tumour were used as negative controls. GFP positive CTCs were then counted using the appropriate gate. Flow cytometric analysis was performed on a FACSCalibur (BD Biosciences, CA).

**Immunohistochemistry assay**

Immunohistochemistry was performed with primary antibodies against IL-17A (Abcam), E-cadherin (Abcam) and N-cadherin (Abcam). The protein expression levels of the target molecules were quantified based on a multiplicative index of staining percentage or extent (0–3) and the staining intensity (0–3) ([Qin et al., 2013](#_ENREF_4); [Yuan et al., 2017](#_ENREF_7)).

For MIH, MAH and paired PVTT (PVTT and matched primary tumour tissues) tissues, the sections were evaluated using light microscopy at 400× magnification. Five representative fields for each case were captured. The IL-17+ cell score was evaluated according to a multiplicative index of the percentage of positive-staining cells (0-3; no staining is scored 0, 50% percentage is scored 1.5, 100% percentage is scored 3; the higher the percentage of positive cells, the higher the score) and the staining intensity (0-3; no staining is scored 0, moderate staining is scored 1.5, strong staining is scored 3; the higher the intensity, the higher the score). The protein expression of E-cadherin and N-cadherin was quantified based on a multiplicative index of staining extent (0–3; no staining is scored 0, 50% area is scored 1.5, 100% area is scored 3; the larger area, the higher the score) and the staining intensity (0–3; no staining is scored 0, moderate staining is scored 1.5, strong staining is scored 3; the higher the intensity, the higher the score). For each case, the final score of the target protein is the average product in five count areas. Quantification of the target molecule expression was performed blindly by a pathologist.

The target protein staining in TMAs was evaluated at 200× or 400× magnification using light microscopy by two investigators blind to the clinical outcome and knowledge of the clinicopathological data. The score of the target molecule in TMAs is same as the criteria for common sections.

**RNA extraction, cDNA synthesis and real-time PCR**

Total RNA were isolated from cell lines as indicated in the figure legends using Trizol reagent (Takara, Dalian, China). The quality of the total RNA was assessed using a Nanodrop 2000 and agarose gel electrophoresis. First-strand cDNA was synthesized from 1 µg of total RNA using random primers and M-MLV Reverse Transcriptase (Invitrogen, CA). Real-time PCR was performed according to the SYBR Green protocol in a StepOne Plus system (Applied Biosystems, Foster City, CA) with β-actin as the endogenous control. The relative expression of RNAs was calculated using the comparative Ct method, and the gene-specific primers are listed in Table S2.

**Western blot analysis**

Proteins were harvested as indicated in the figure legends, separated by sodium dodecyl sulphate polyacrylamide gel electrophoresis and transferred onto nitrocellulose filter membranes. After incubation with antibodies specific for E-cadherin (Abcam), ZO-1 (Abcam), N-cadherin (Abcam), vimentin (Cell Signaling Technology, Boston, USA), snail (Abcam), slug (Abcam), twist1 (Abcam), ZEB1 (Abcam), ZEB2 (Abcam), AKT (Cell Signaling Technology), phospho-AKT (Cell Signaling Technology) or β-actin (Sigma-Aldrich), the blots were incubated with IRdye 800-conjugated goat anti-rabbit IgG and IRdye 700-conjugated goat anti-mouse IgG and were detected using an Odyssey infrared scanner (Li-Cor). β-actin was used as a loading control for western blots.

**Immunofluorescence analysis**

The cells were incubated in 24-wells plate with cover-slips for 24 h. After fixing in 4% paraformaldehyde for 15 minutes at room temperature, cells were blocked with 5% BSA at room temperature for 60 min. The cells were first incubated with antibodies specific for IL-17R (Abcam), E-cadherin (Abcam), ZO-1(Invitrogen), N-cadherin (BD Transduction Laboratories) or vimentin (Cell Signaling Technology), and then with goat anti-rat IgG (Alexa Fluor 594, Invitrogen), goat anti-mouse IgG (Alexa Fluor 488, Invitrogen) or goat anti-rabbit IgG (Alexa Fluor 594, Invitrogen). The slides were mounted by adding DAPI-Fluoromount-G (Southern Biotech, SBA, Birmingham, AL) and examined with a Zeiss Axiophot photomicroscope (Carl Zeiss, Oberkochen, Germany).

**Isolation of CD4+ T Cells and Th17 Cell Culture**

Buffy coats from the healthy donors were processed to purify peripheral blood mononuclear cells (PBMCs). The human CD4+ T cell isolation kit-II (Miltenyi Biotec, Paris, France) was used to isolate untouched total CD4+ T cells by negative selection. After CD4+ T cell isolation as completed, the cells were counted with a haemocytometer and kept on ice. Then, 1.5 mL of 2x mix of Th17-polarising medium was added to each well containing anti-human IFNγ (20 µg/mL), anti-human IL-4 (20 µg/mL), human TGFβ (10 ng/mL), human IL-1β (40 ng/mL), human IL-23 (40 ng/mL), and human IL-6 (50 ng/mL) all diluted in a serum-free base medium (supplemented with 2 mM L-glutamine, 100 U/mL penicillin, 100 µg/mL streptomycin, 10 mM HEPES, 1 mM sodium pyruvate and 100 µM 2-mercaptoethanol). After 3-days of culturing and stimulation, the cells were harvested to investigate the Th17 differentiation status by measuring IL-17A (BD) expression using a flow cytometer (LSRFortessa, BD). To test the influence of Th17 cell manipulation in HCC cell EMT, 10 ml of fresh solution harvested from the culture medium of the Th17 cells incubated for72 h was concentrated into 0.1 ml using an ultrafiltration tube (Millipore) with a molecular weight cut-off of 3 kD. Then, the concentrated supernatants were added into the conditioned medium from HCC cells at a ratio of 1:2.

**Enzyme-linked immunosorbent assays (ELISA)**

The levels of IL-17A, IL-6 in culture supernatants were measured by ELISA, following the manufacturer’s instructions (R&D Systems).

**Supplemental References**

Huang, G., Lau, W.Y., Zhou, W.P., Shen, F., Pan, Z.Y., Yuan, S.X., Wu, M.C., 2014. Prediction of Hepatocellular Carcinoma Recurrence in Patients With Low Hepatitis B Virus DNA Levels and High Preoperative Hepatitis B Surface Antigen Levels. JAMA surgery 149, 519-527.

Huang, G., Yang, Y., Shen, F., Pan, Z.Y., Fu, S.Y., Lau, W.Y., Zhou, W.P., Wu, M.C., 2013. Early viral suppression predicts good postoperative survivals in patients with hepatocellular carcinoma with a high baseline HBV-DNA load. Annals of surgical oncology 20, 1482-1490.

Ma, S., Ling, F., Gui, A., Chen, S., Sun, Y., Li, Z., 2017. Predictive Value of Circulating Tumor Cells for Evaluating Short- and Long-Term Efficacy of Chemotherapy for Breast Cancer. Medical science monitor : international medical journal of experimental and clinical research 23, 4808-4816.

Qin, J., Wu, S.P., Creighton, C.J., Dai, F., Xie, X., Cheng, C.M., Frolov, A., Ayala, G., Lin, X., Feng, X.H., Ittmann, M.M., Tsai, S.J., Tsai, M.J., Tsai, S.Y., 2013. COUP-TFII inhibits TGF-beta-induced growth barrier to promote prostate tumorigenesis. Nature 493, 236-240.

Tao, Q.F., Yuan, S.X., Yang, F., Yang, S., Yang, Y., Yuan, J.H., Wang, Z.G., Xu, Q.G., Lin, K.Y., Cai, J., Yu, J., Huang, W.L., Teng, X.L., Zhou, C.C., Wang, F., Sun, S.H., Zhou, W.P., 2015. Aldolase B inhibits metastasis through Ten-Eleven Translocation 1 and serves as a prognostic biomarker in hepatocellular carcinoma. Molecular cancer 14, 170.

Yang, Y., Liu, Y.M., Wei, M.Y., Wu, Y.F., Gao, J.H., Liu, L., Zhou, W.P., Wang, H.Y., Wu, M.C., 2010. The liver tissue bank and clinical database in China. Frontiers of medicine in China 4, 443-447.

Yuan, J.H., Liu, X.N., Wang, T.T., Pan, W., Tao, Q.F., Zhou, W.P., Wang, F., Sun, S.H., 2017. The MBNL3 splicing factor promotes hepatocellular carcinoma by increasing PXN expression through the alternative splicing of lncRNA-PXN-AS1. Nature cell biology 19, 820-832.
